# Neuropathology and Animal Models of Autism: Genetic and Environmental Factors

**DOI:** 10.1155/2013/731935

**Published:** 2013-09-16

**Authors:** Bharathi S. Gadad, Laura Hewitson, Keith A. Young, Dwight C. German

**Affiliations:** ^1^Department of Psychiatry, University of Texas Southwestern Medical Center, Dallas, TX 75390-9070, USA; ^2^The Johnson Center for Child Health and Development, Austin, TX 78701, USA; ^3^Psychiatry and Behavioral Science, Texas A&M Health Science Center, Temple, TX 76504, USA

## Abstract

Autism is a heterogeneous behaviorally defined neurodevelopmental disorder. It is defined by the presence of marked social deficits, specific language abnormalities, and stereotyped repetitive patterns of behavior. Because of the variability in the behavioral phenotype of the disorder among patients, the term autism spectrum disorder has been established. In the first part of this review, we provide an overview of neuropathological findings from studies of autism postmortem brains and identify the cerebellum as one of the key brain regions that can play a role in the autism phenotype. We review research findings that indicate possible links between the environment and autism including the role of mercury and immune-related factors. Because both genes and environment can alter the structure of the developing brain in different ways, it is not surprising that there is heterogeneity in the behavioral and neuropathological phenotypes of autism spectrum disorders. Finally, we describe animal models of autism that occur following insertion of different autism-related genes and exposure to environmental factors, highlighting those models which exhibit both autism-like behavior and neuropathology.

## 1. Introduction

**Figure 1:**
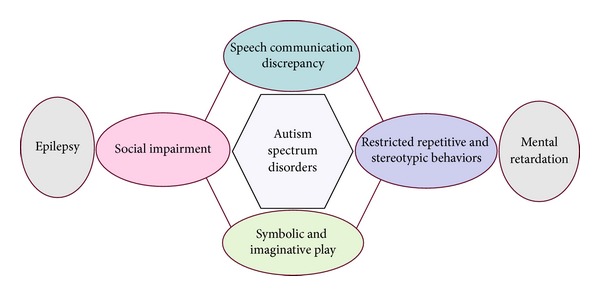
Symptoms of autism as per DSM-IV criteria. The core symptoms of autism are related to speech and language problems, stereotyped and repetitive behaviors, and social impairment. Other symptoms often associated with ASDs are epilepsy and mental retardation.

 Autism is a heterogeneous neurodevelopmental disorder with multiple causes and a great range in the severity of symptoms [1, 2]. As described by Kanner in 1943, individuals with autism have four core features: (i) impairments in reciprocal social interactions, (ii) an abnormal development in the use of language, (iii) repetitive and ritualized behaviors, and (iv) a narrow range of interests [3]. These symptoms range from mild to severe as defined in the Diagnostic and Statistical Manual of Mental Disorders, Fourth edition (DSM-IV) [4] (Figure 1). In addition to the core features, people with autism often have comorbid neurological disorders such as mental retardation and epilepsy [5]. The prevalence of mental retardation with autism is ~60%, but in the broader autism spectrum disorders (ASDs), the number is closer to 30% [6]. Epilepsy has been long associated with autism although estimates of the occurrence of seizure disorder vary from 5% to 44% [7]. Anxiety and mood disorders are very common in autism [8]. There is also a substantial heterogeneity in the onset of autism. Impairments in some children manifest before 18 months of age; however, 25%–40% of children with autism initially demonstrate near normal development until 18–24 months, when they regress into autism that is generally indistinguishable from the early onset form of the disorder [8]. The early onset versus regressive phenotypes of autism suggest different neuropathological mechanisms.

 Neuropathological observations that have emerged over the past decade point towards early pre- and postnatal developmental abnormalities that involve multiple regions of the brain, including the cerebellum, cortical white matter, amygdala, brain stem, and cerebral cortex. However, since 1980, only 120 postmortem brains from people with autism have been studied [9]. Thus, the neuropathology literature is neither extensive nor rigorous, and there are several areas that remain open to further investigation. In the present review, we have highlighted neuropathological features of the areas that may play an important role in the pathology of autism.

ASDs constitute a diverse set of symptoms with multiple etiologies including genetic susceptibility and interactions between genetic and environmental factors. Because of the wide range of potential environmental factors thought to contribute to autism, well-defined animal models that can display core symptoms, neuropathological, and behavioral features are essential for autism research. Many studies indicate that genetic factors play a role in at least half of the cases of autism. In identical twins, if one twin has autism, over half of the other twin siblings will also exhibit autism [10, 11]. The first genes implicated in autism were associated with broader syndromes that included autistic symptoms; genes associated with tuberous sclerosis (TSC1 and TSC2) and the tumor suppressor gene PTEN show associations with autistic symptoms [12]. The monogenic disorders Rett syndrome and Fragile X syndrome lead to stereotyped repetitive hand movements and a regression of neurological and social skills in some of the carriers [13, 14]. Mutations in the X-chromosome linked genes neuroligin 3 and 4 can cause autism, mental retardation, and other neuropsychiatric syndromes [15]. Rare variants of the contactin-associated protein-like 2 (CNTNAP2) gene can increase the risk of developing autism [16]. It is clear that there is no “autism gene”. Rather, it has been estimated that between 350 and 400 autism susceptibility genes exist, and many of which are associated with the Fragile-X mental retardation protein (FMRP), based upon exome sequencing studies of families [17]. Many of these mutations are thought to be *de novo* and occur prior to conception, often in the paternal line. Thus, multiple genetic variants appear to interfere with brain development and cause ASDs [18].

Several genetic models of autism have been developed in mice that may provide insight into underlying causes for the disorder. However, while genetic factors play a strong role in ASDs, environmental factors like thalidomide, valproic acid, thimerosal, maternal infection, and vaccines may also contribute to the complex pathogenesis of ASDs. In the present review, we will examine the neuropathology and neuroanatomy of ASDs and the genetic and environmental factors that both contribute to ASD-like neuropathology and underlie animal models of the disorder.

## 2. Neuropathology of Autism

 Neuropathological studies on autism have reported reductions in cell number and cell size in the cerebellum, limbic system, brainstem, cortex, amygdala, and hippocampus. However, it should be noted that many areas of the brain have never been studied, and so this list should not be interpreted as an indication that selective changes occur in these regions alone in autism.  Table 1 summarizes the neuropathology observations in postmortem autism brains since 2003, updating data previously published [19]. The following brain regions have been found to exhibit neuropathology in ASD cases.

### 2.1. Cerebellum

Multiple studies have found neuronal abnormalities in the cerebellum in postmortem brains from people with ASDs [21, 22]. Structural neuroimaging investigations have shown decreases in both cerebellar gray and white matters [23, 24]. Fatemi and colleagues [23] showed a 24% decrease in mean Purkinje cell size in 5 autistic brains. The Purkinje cell is the output neuron of the cerebellum, and it uses the inhibitory neurotransmitter, GABA. Two of the five autistic subjects had greater than a 50% reduction in Purkinje cell size compared to 5 control cases. Bailey and colleagues [25] reported Purkinje neuron reductions in 5 autism cases versus 7 control cases. Of these, 3 demonstrated a diffuse reduction, while 2 showed greater reduction in the hemispheres than the vermis, consistent with findings of Bauman and Kemper [26]. In contrast, Lee and colleagues reported on two cases with Purkinje neuron reductions, one of which showed greater reduction in the vermis than in the hemispheres [27]. All of these data, however, are semiquantitative, so before firm conclusions can be drawn about the cerebellum and autism, unbiased quantitative methods will need to be applied. In a recent stereological study of 6 ASD and 4 control cases, reductions in Purkinje cell number were found only in 3 of 6 ASD cases [28], indicating variability in the neuropathological data. Also, the enzyme used to synthesize GABA has been found to be reduced by >50% in dentate gyrus neurons (a deep cerebellar nucleus) to which the Purkinje cells communicate in 5 ASDs versus 5 normal control postmortem brains [29].

Autopsy studies have provided interesting clues regarding the timing of Purkinje cell loss during development. The absence of reactive gliosis and the lack of empty basket cells, which normally ensheath the Purkinje cell bodies, have provided suggestive evidence for a prenatal reduction of Purkinje cells [30, 31]. This is also supported by the absence of atrophy of the cerebellar folia [25]. On the other hand, Bailey and coworkers also reported increased numbers of Bergmann glia in some of their autopsy cases, in addition to increased glial fibrillary acidic protein, which point to a later stage for pathology to begin. It may be that Purkinje neurons degenerate during the postnatal period in some cases of ASDs and prenatally in others. 

**Figure 2:**
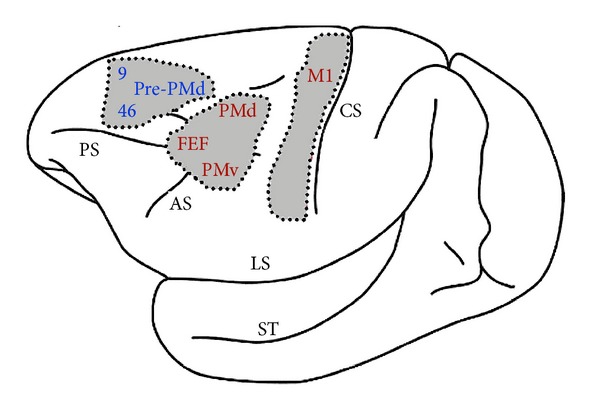
The cerebellum projects to motor and nonmotor regions in the monkey brain. Targets of cerebellar output, indicated in red, are areas of the cerebral cortex that have motor-related functions. Blue labels indicate cortical areas that are the nonmotor targets of cerebellar output. The areas are indicated on the lateral aspect of the Cebus monkey brain. The numbers 9 and 46 refer to cytoarchitectonic areas of the prefrontal cortex. AS: arcuate sulcus; FEF: frontal eye field; LS: lateral sulcus; M1: areas of the primary motor cortex; PMd: dorsal premotor area; PMv: ventral premotor area; PrePMd: predorsal premotor area; ST: superior temporal sulcus. Modified from Strick et al. [20].

Neurodegeneration of cerebellar Purkinje cells is not found in all brains from people with ASDs, but there is considerable evidence that the cerebellum can be a major neuropathological target. Although people with ASDs do not present typical motor signs of cerebellar dysfunction, the cerebellum is involved with many nonmotor functions such as cognition, language, imitation, attention, and mental imagery due to its projections to nonmotor regions of the cortex and limbic system [20, 21, 31–35]. As illustrated in Figure 2, the cerebellum projects not only to several motor-related areas of the cortex (e.g., motor cortex, premotor cortex, and frontal eye fields), but also to the frontal cortex (e.g., areas 9 and 46), which supports cognitive functions. Cerebellar damage in children is often associated with an autism phenotype, as reviewed by Allen [21, 22]. Finally, selective genetic knockout (KO) of the Tsc1 gene in mouse cerebellar Purkinje cells reproduces several autism behavioral phenotypes [36]. These animals display Purkinje cell dysfunction and subsequent degeneration, accompanied by abnormal social interaction, repetitive behaviors, and repetitive vocalizations. Interestingly, chronical treatment with rapamycin, the mTOR inhibitor, blocked the behavioral and neuropathological effects of the transgene insertion in such animals.

### 2.2. Limbic System

In addition to the cerebellum, the limbic system has been implicated in the pathophysiology of autism. The limbic system plays a role in learning, social functioning and emotion, functions that are typically disturbed in autism. Neuropathological studies of the limbic system in autism have found decreased neuronal size, increased neuronal packing density, and decreased complexity of dendritic arbors in the hippocampus, amygdala, and other limbic structures [43, 44].

#### 2.2.1. Amygdala

The amygdala plays an important role in the mediation of social behavior, facial and emotional recognition, enhancement of memory for emotionally significant events, and prediction of reward values [37, 45, 46]. For this reason, it is one of the structures most thoroughly studied in autism. Here, the amygdala undergoes an abnormal developmental time course that includes a precocious enlargement that persists through late childhood. Several studies have shown a 13–16% enlargement of the amygdala in young autistic cases (3–5 years of age) [37, 46]. Schumann and colleagues [45] found that although the amygdala grows by 40% between 8 and 18 years of age in typically developing boys, autistic boys exhibit a stagnation of growth. Thus, even though the amygdala volume differs significantly in the younger ages between ASDs and controls, the volume normalizes in the adolescent and adult age groups because of different growth patterns between the two groups. A significant correlation was found between the severity of clinical presentation at ~5 years of age and amygdala enlargement at 3 years of age. Recent studies suggest that amygdala enlargement is associated with elevated anxiety and poor social and communication skills [45, 46]. At the microstructural level, Schumann and Amaral [37] studied several amygdala subnuclei as well as the entire nucleus in brains from adolescent to young adults (range 10–44 years). Using stereological methods, they found a significant 14% decrease in total neuron number in the lateral nucleus and a significant 12% decrease in neuron number in the total amygdala in 9 autism brains versus 10 age-matched typically developing control brains. Aylward and colleagues [47] reported that amygdala volume was significantly smaller in the autistic subjects versus age-matched controls (*n* = 14/group) based on MRI imaging studies in adolescents and adults. Thus, the amygdala size and cell number differ in ASDs versus controls, but the difference depends upon the age of the ASD subject.

#### 2.2.2. Hippocampus

The hippocampus plays a pivotal role in memory consolidation and retrieval. Several studies have found abnormalities in this brain structure in ASD subjects. Aylward and colleagues [47] reported that hippocampal shape is significantly different in autistic subjects versus age-matched controls, and Schumann and colleagues [46] found that children with ASDs had a larger right hippocampal volume versus typically developing children. Earlier, Kemper and Bauman [44] reported increased cell packing density and smaller neurons in the CA1 region of hippocampus in all autistic cases examined (*n* = 6 autistic versus 6 age- and gender-matched controls), using semiquantitative methods. How these changes in hippocampal structure impact the ASD phenotype is unclear.

### 2.3. Cerebral Cortex

Autism is a disorder that markedly affects executive function and high-order integration processes such as complex social interactions, associative thinking, and appropriate emotional reactions. This has led some researchers to focus on cortical brain regions associated with these functions: the frontal cortex and fusiform gyrus. 

#### 2.3.1. The Frontal Cortex

Brain imaging studies indicate that in the first year of life the frontal cortex, specifically its dorsolateral and medial parts, grows disproportionally larger in volume than the rest of the cortex in individuals with ASDs. Afterwards, from 2 to 9 years of age, these cortical regions show a volume enlargement of only 10% in autistic children versus 48% enlargement in the control group (*n* = 25 males/group) [48, 49]. Courchesne and colleagues [40] reported more neurons (67%) and increased brain weight (17.6%) in the dorsolateral and medial prefrontal cortices in autistic versus control children (7 ASD; 6 controls, aged 2−16 years); Santos and colleagues [41] also found more neurons (58%) in autism versus control children (4 autism; 3 controls aged 4–11 years) in the fronto-insular cortex, and Jacot-Descombes [42] observed smaller pyramidal neuronal size (18%) in Brodmann areas 44 and 45 in autism versus controls (8 autism; 8 controls 4–66 years) and no change in cell number in the dorsolateral prefrontal cortex (see Table 1). These Brodmann areas, as well as the fusiform gyrus, (see below) contain the socalled mirror neurons associated with mimicry, a behavior which may underlie the development of communication and language, making alterations in this area of the cortex interesting from a theoretical viewpoint.

#### 2.3.2. Fusiform Gyrus

The fusiform gyrus, in the temporal lobe, is involved in face processing. A neuropathological study, using rigorous stereologic methods, reported significant reductions in neuron density in layer III (13%) and in neuron number in layers III, V, and VI (14%), as well as decreased pyramidal cell volume in layers V and VI [38]. In this study, 7 subjects with autism (mean age = 12 years) and 10 controls (mean age = 30 years) were analyzed, and the age difference between ASD and control subjects was not shown to be a factor in the results. The fusiform gyrus has been examined using PET imaging of “activated microglia” with [^11^C] (*R*)-PK11195. There was a significantly enhanced microglial activation in young adult ASD male brains versus age- and IQ-matched control males (*n* = 20/group). In addition to the fusiform gyrus, there was also enhanced activation in the cerebellum, the anterior cingulate, and orbitofrontal cortices [50].

### 2.4. White Matter

Studies of cerebral white matter in people with ASDs indicate changes in organization, maturation rate, and structural integrity [51]. Diffusion tensor imaging is an advanced MRI technique that enables the measurement of the diffusion of water in tissue in order to allow the direction and packing density of fiber tracks to be estimated. To assess the fiber density, axonal diameter, and myelination stage, a parameter called fractional anisotropy (FA) is measured. In a recent study by Shukla and colleagues [52], structural changes in FA were observed in several cortical regions involved in social cognition and information integration using 26 ASD and 24 typically developing participants aged 9–20 years. Noriuchi and colleagues [53] also found an excess of white matter in the cerebellar vermis lobule, a region known for its abnormal cytoarchitecture in autism in high-functioning ASD cases (*n* = 7 versus 7 control subjects aged 13 ± 3 years).

### 2.5. Cortical Spine Densities

Hutsler and Zhang [39] studied pyramidal cell dendritic spine densities in 10 autism and 15 control cortices using classical Golgi methods. They observed consistent evidence for increased densities of spines in all layers and in all 3 brain areas investigated. The most robust evidence for increased densities was observed in layer 2 of BA9 (superior frontal), BA7 (parietal), and BA21 (temporal), and in layer 5 of BA21. Increased spine densities were observed in the autism cases across the entire age range investigated, from 10 to 46 years. It is interesting to note that elevated densities of spines also have been observed in Fragile X syndrome, an ASD [54]. The authors note that the localization of increased densities in layer 2 is interesting because unlike other layers studied, this lamina does not develop spines until the postnatal period. The data suggest that the anatomical pathology of autism may involve abnormal spine generation or deficits in spine reorganization, elimination, and pruning. This cellular process may contribute to the observations of amygdala and cortical overgrowth in early development, which may be signs of deficits in cortical and amygdala spine reorganization and consolidation.

Thus, classical neuropathological and neuroimaging studies report substantial evidence for cerebellar changes in autism such as decreased numbers of Purkinje cells. There is also an interesting pattern of early developmental overgrowth, increased cell packing density, and smaller neuronal size in the limbic cortex and amygdala, followed by stunted growth that lasts through adolescence. This abnormal developmental pattern may be driven by core deficits in reorganization and consolidation of dendritic spines. Overall, these findings suggest that early developmental insults, produced by genetic and/or environmental factors, may alter normal brain development in autism during this critical period when social and cognitive skills are being learned.

## 3. Animal Models of Autism

In this section of the review, we discuss animal models of autism that have been linked to genetic and environmental factors. We focus on those models that exhibit neuropathological and behavioral endophenotypes that are linked to ASDs and that impact the immune system. The endophenotypes include seizures, anxiety, aggressive behavior, gastrointestinal problems, motor deficits, abnormal sensory processing, and sleep disturbances (see review [55]).

### 3.1. Genetic Factors

Six autism-related genes, linked to the X-chromosome, have been identified in autism. These genes are the Fragile X mental retardation gene (Fmr1), methyl-CpG-binding protein type 2 gene (MECP2), neuroligin (NLGN) 3 and 4 genes, and tuberous sclerosis genes (TSC1 and TSC2). In addition, mutations in the DLX, Reelin, Engrailed, and PTEN genes also result in autism phenotypes and neuropathology. The following section describes genetic mouse models that exhibit autism-like behaviors, neuropathology, and immune system alterations. 

#### 3.1.1. Fmr1

Approximately 30% of children with Fragile X syndrome share a number of symptoms in common with autism, such as mental retardation, attention deficit hyperactivity disorder, and epilepsy [56–58]. Fmr1 KO mice display abnormally long and thin dendritic spines of layer V pyramidal neurons in the cerebral cortex [59, 60]. These animals show decreased active social behaviors when confronted with wild-type control mice, reduced trace fear conditioning and altered social interactions [59–61]. FMR1 KO mice exhibit elevated cortical spine densities similar to those observed in autism and Fragile X syndrome [62]. 

#### 3.1.2. MeCp2

The MeCp2 gene is related to Rett syndrome, a genetic disorder that is currently considered one of the ASDs. In mouse models with MeCp2 disruption, the animals are normal until about 16 weeks of age (a mouse typically is mature by 4 weeks and dies at 2-3 years) after which they exhibit enhanced anxiety in the open field, reduced nest building, and aberrant social interactions [63]. Restoring MeCp2 expression, in a conditional KO model, results in reversal of the disease phenotype. The MeCp2 KO mouse shows overtly normal development for about the first month of life, followed by increasingly severe neurological abnormalities, and death by approximately 10 weeks of life. The mutant behavioral phenotype includes hypoactivity, seizure-like responses, and stereotyped forelimb movements, body trembling, gait ataxia, and limb clasping reminiscent of the repetitive hand wringing observed in some children with ASDs [64, 65]. Mice with Rett KO specifically within GABAergic neurons exhibit ASD-like behavior, suggesting that the GABA neurons are an important target for behavioral abnormalities [66]. Recently, Yang and colleagues [67] reported that helper T-cells from children and mice with MECP2 duplication displayed similar reductions in interferon-*γ* secretion potentially leading to a partially immunodeficient state, implicating the immune system as playing a role in autism.

#### 3.1.3. Neuroligin

The NLGN 3 and 4 genes map to three loci associated with predisposition to autism, 3q26, Xp22.3, and Xq13, respectively [68]. Mutations in NLGN 3 and 4 are associated with autism and, in some cases, with mental retardation, a feature often associated with autism. Recently, four novel synonymous substitutions in the X-linked genes NLGN3 and NLGN4X have been reported in a Japanese with ASDs [69], and the mutations are more common in males [70]. Comoletti and colleagues [68] found that these NLGN3 and 4 mutations lead to loss of neuroligin processing for stimulating the formation of synapses. NLGN3 KO mice exhibit disrupted heterosynaptic competition and perturbed metabotropic glutamate receptor-dependent synaptic plasticity, a hallmark feature of Fragile X and ASDs [71]. Such changes in synaptic connections may be the most common, fundamental feature of all people with ASDs.

#### 3.1.4. Tuberous Sclerosis

Tuberous sclerosis (TSC) is a genetic disease sometimes associated with autism-like symptoms in which mutations in one of two TSC genes cause multiple, benign tumors to grow in various tissues including the brain [72]. There is a high incidence of autism-like symptoms in TSC. The cerebellum is abnormal in many children with tuberous sclerosis [73], and there is a positive correlation between the magnitude of cerebellar pathology and ASD symptomatology [74, 75]. Hamartin and tuberin, the protein products of TSC1 and TSC2, inhibit the mammalian target of rapamycin (mTOR) [72]. Recently, Tsai and colleagues [36] observed autistic-like behaviors in mice expressing mutant TSC1 specifically in cerebellar Purkinje cells. In these mice, the size of the Purkinje cell significantly increased prior to their death. During this enlargement, the cells fired action potentials at markedly reduced rates. The animals exhibited autism-like behavioral deficits such as abnormal social interactions, repetitive behaviors, and vocalizations. This is an interesting study because it demonstrates that the behavioral phenotype of autism occurs following selective changes in cerebellar output (i.e., the Purkinje cells).

#### 3.1.5. DLX

The DLX genes have been associated with autism [76], and they regulate the development of a subset of cortical and striatal neurons. Two of the linkage loci for autism, 2q31.1 and 7q21.3, contain the DLX1/2 and DLX5/6 complexes, respectively. Stühmer and colleagues [77] reported that mutations in the DLX2 and 5 genes alter the development of GABA neurons in the forebrain. As mentioned above, the GABAergic system (e.g., cerebellar Purkinje cells use the neurotransmitter GABA) can be involved with the neuropathology of autism, and genetic mutations that cause GABAergic insufficiency also cause autism-like behaviors [78], as well as seizure activity which often accompanies ASDs.

#### 3.1.6. Reelin

The Reelin gene, located on chromosome 7, has been linked to autism [79]. In the adult brain, Reelin is normally expressed in GABAergic neurons [80]. The importance of the cerebellum is again suggested because Reelin regulates dendritic sprouting in the GABAergic cerebellar Purkinje cells [81, 82]. In the heterozygous Reelin mouse, there is a 16% loss of Purkinje cells in 3-month-old mice and 24% loss by 16 months of age [83]. Furthermore, the Purkinje cell loss is observed mainly in male animals while the females are spared, suggesting that the Reelin gene exerts its effect on Purkinje cell number in a gender-specific fashion, mirroring the increased male incidence of ASDs. These mice exhibit dissociations between social task performance and reversal learning, consistent with an autism-like behavioral phenotype.

#### 3.1.7. Engrailed

The gene Engrailed 2 (En2) is located on chromosome 7 and has been linked to autism. Mouse variants of En2 and autistic individuals display similar cerebellar morphological abnormalities [84]. En2 KO mice show a 10–12% loss of Purkinje cells. Other features shared between the En2 KO mice and people with autism are deficiencies in the number of deep nuclear, granule, and inferior olive neurons [25]. Increased neuronal packing, a smaller hippocampus, and ectopic location of neuronal subgroups in the amygdala in En2 KO mice also have been observed in autism postmortem brains [25, 84].

#### 3.1.8. PTEN

PTEN (phosphatase and tensin homolog deleted on chromosome 10) germline mutations are found in a small subset of children diagnosed with ASDs with accompanying macrocephaly. The first study clearly linking PTEN mutations to autism examined the PTEN gene in 18 individuals with autism and macrocephaly and found that 3 individuals (17%) carried germline mutations [85]. Mice with PTEN loss, limited to postmitotic neurons in the hippocampus and cortex exhibit loss of neuronal polarity and macrocephaly [86, 87]. The mutant mice exhibit behavioral abnormalities reminiscent of certain clinical features of autism, such as anxiety, decreased social interest and seizures. These defects were reversed by treatment with rapamycin, implicating the mTOR pathway downstream of PTEN as critical for the neuronal and behavioral phenotypes. PTEN is also involved in dendritic spine pruning [88], so loss of function via PTEN mutations may impair normal synaptic plasticity and contribute to the increased spine density observed in postmortem ASD brains.

In review, animal models using genetic targets identified from human studies of autism have provided evidence that supports the involvement of the cerebellum and more specifically, GABAergic neurotransmission in the pathophysiology of the disorder. These GABAergic changes may represent a final common pathway for autistic behaviors. Evidence for this possibility comes from studies indicating that autism-like behaviors associated with knock-down of non-GABAergic products such as Scn1a can be rescued by altering GABA neurotransmission [78]. Also worth noting is the involvement of several genes that appear to affect synaptic plasticity in a way that could result in accentuated numbers of spines, as observed in autism. As the neuropathology of autism becomes better described, these animal models will become even more important in selecting cellular pathways that could be targeted for treatment or prevention.

### 3.2. Environmental Factors

Environmental factors potentially contributing to developmental disorders have been studied in animal models and have been observed to influence brain development and to play a role in CNS neuropathology. 

#### 3.2.1. Thalidomide and Valproic Acid

Thalidomide, an anti-nausea drug used by pregnant women between 1957 and 1962 was shown to be linked to a marked increase in the incidence of autism in their offspring [89]. Several reports have linked serotonin to autism [90, 91], and in rats, thalidomide exposure at embryonic day 9 (E9) causes increased plasma, hippocampal, and frontal cortex serotonin. Abnormal development of the serotonin system in this animal model implicates agents that alter serotonin in early development as possible environmental contributors to autism. Of the environmental agents linked to autism, valproic acid (VPA) has been studied most extensively. VPA is an anticonvulsant and mood-stabilizing drug, primarily used for the treatment of epilepsy and treatment-resistant depression. As with thalidomide, VPA exposure on E9 causes hyperserotonemia in the mouse hippocampus, frontal cortex, and cerebellum [92]. VPA enhances DNA demethylation, and while this mechanism may be useful for reverse hypermethylation in epilepsy and depression, it may interfere with methylation processes necessary for normal brain development. The offspring of women taking VPA during early pregnancy have an increased risk of autism [93]. The offspring of pregnant rats given VPA show an 11% reduction in cerebellar Purkinje cells and decreased cerebellar volume (31%). In addition, the number of neurons in the inferior olive, providing input to Purkinje cells, is also significantly reduced (9%) [94]. 

#### 3.2.2. Mercury

Methyl mercury exposure during childhood is associated with neuropsychological abnormalities in language, attention, and memory [95–97]. DiCicco Bloom and coauthors reported that acute methyl mercury exposure during development elicits hippocampal cell death, reductions in neurogenesis, and severe learning deficits [95]. A single injection of methyl mercury in 7-day-old rats resulted in reductions in hippocampal size (21%) and cell number two weeks later, especially in the granule cell layer (16%) and hilus (50%) of the dentate gyrus. In humans, such exposure levels can come from eating whale blubber, as in people who live in the Faroe Islands, or from living in an environment contaminated by mercury.

Thimerosal is a mercury-containing organic compound (an organomercurial). It has been widely used since the 1930s as a preservative in a number of biological and drug products, including many vaccines, to help prevent microbial contamination. Thimerosal contains ethyl mercury, which some parents have suspected to be associated with adverse neurodevelopmental outcomes, including autism [98]. The association between exposure to thimerosal-containing vaccines and developmental outcomes has been debated since 1999 when the Food and Drug Administration determined that children who received multiple thimerosal-containing vaccines at a young age were at risk for exceeding the Environmental Protection Agency's safety limits for methyl mercury [99]. Several epidemiological studies have sought to determine whether childhood vaccines containing thimerosal result in neurodevelopmental disorders including autism; however, both significant and nonsignificant associations have been reported [99–101]. 

A relationship has been reported between thimerosal-containing vaccines and tics in several studies. Thompson and colleagues [99] investigated the association between the receipt of thimerosal-containing vaccines and immune globulins early in life on neuropsychological outcomes in children at 7–10 years of age. The data included the evaluation of 1,047 children and their biological mothers with 24 neuropsychological tests. The only variable that was statistically significant was tics that is, children that were exposed to higher doses of thimerosal were more likely to exhibit tics. In a follow-up study by Barile and coworkers [102], examining a subset of the data examined by Thompson and colleagues [99], they found a significant association between thimerosal dosage and tics, but only in boys. They found no statistically significant associations between thimerosal exposure from vaccines early in life and six of the seven neuropsychological constructs. Two additional studies have also found a relationship between tics and thimerosal exposure [103, 104]. A study by Tozzi and colleagues [105] did not find a relationship between thimerosal dosage and tics but did find a relationship between thimerosal exposure and lower finger-tapping scores and Boston Naming Test scores in girls.

#### 3.2.3. Maternal and Paternal Factors

A large epidemiological study using the Danish Medical Register recently indicated that maternal infection is a risk factor for autism in the offspring. An examination of 10,000 autism cases found a significant association with maternal viral infection in the first trimester [106]. To test this association in animals, rodents were exposed to maternal immune activation with polyinosine:cytosine (poly I:C) [107–109]. When given at embryonic day 9.5, the offspring displayed histological and behavioral abnormalities that resembled autism. Behaviorally, these rodents exhibited communication differences, decreased sociability, and increased repetitive stereotyped behaviors and had smaller brain sizes at birth, followed by macroencephaly in adulthood. With respect to brain neuropathology, the offspring of maternally infected mice displayed significantly fewer (7%; *P* < 0.05) Purkinje cells but only in Lobule VII. These data are quite similar to both ASD behavioral and neuropathological phenotypes.

 In a recent study, Kong and colleagues [110] found that sperm from older men contain more DNA mutations compared to sperm from young men, and these mutations are commonly found in their autistic offspring. Hence a father's age appears to be a risk factor for disorders such as ASDs. An association between autism and the mother's age was also observed, but the effect seems to be enhanced in the paternal line.

**Figure 3:**
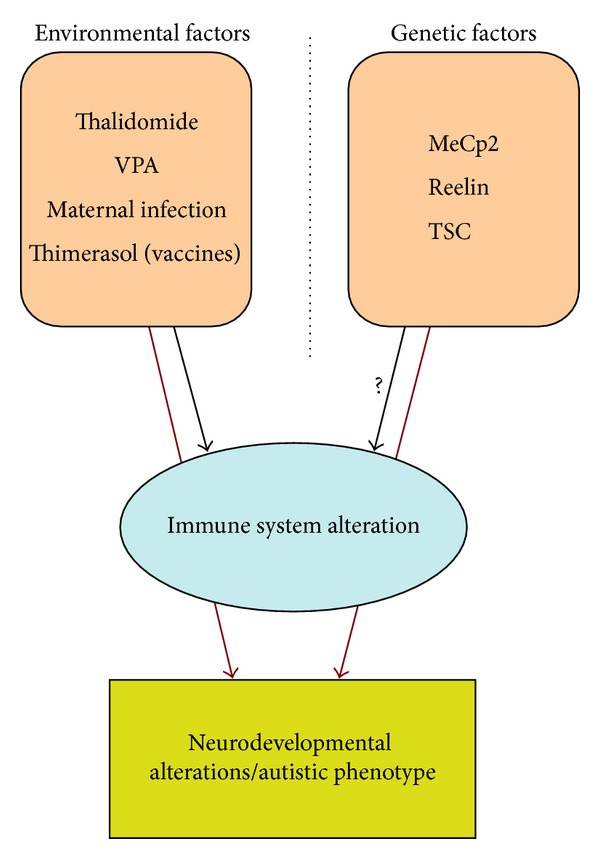
Environmental and genetic factors play a role in autism and altering the immune system. Environmental factors, like valproic acid, thalidomide, and thimerosal-containing vaccines, play a role in causing neurodevelopmental disorders and altering immune system function in humans with autism and in animal models of autism. Genetic mutations in MeCp2, TSC, and *Reelin* can induce an autism phenotype as well as autism-like neuropathology and/or immune system impairment when inserted into mice. The “?” indicates missing evidence for TSC and Reelin causing immune system alterations.

Viral infections in the mother can induce immune system alterations in both mother and fetus, which can lead to long-term epigenetic changes in the offspring [111]. The timing of immune insults during development may be one source of the heterogeneity in the phenotypes observed in ASDs (see Figure 3). For example, (i) thalidomide/VPA treated male rats exhibit immunological alterations such as lower thymus weight, decreased splenocyte proliferative response to mitogenic stimulation, and decreased interferon (IFN)-*γ*/IL-10 ratio in peritoneal macrophages [112]. Female rats in this study did not exhibit many of the behavioral and immunological alterations, suggesting sex-specific responses to some environmental factors. The exaggerated immunological response of males may contribute to the 2 : 1 ratio of male-female diagnosis for ASDs. (ii) Following viral infection, the immune response leads to the production of various cytokines, such as interleukins (IL)-1, -2 and -6 which influence the release of monoamines such as serotonin in the hippocampus and other brain regions [113]. (iii) Maternal infection alters the peripheral immune system, which originates during fetal development. Maternal infection leads to elevated levels of cytokines and chemokines including interleukin-1*β* (IL-*β*), IL-6, IL-8, and IL-12p40 in the plasma of children with ASDs, and such increases are associated with more impaired communication and aberrant behaviors [114]. The offspring of infected rodent mothers given poly (I:C) exhibit both ASD-like behavioral and neuropathological abnormalities as described above [107–109, 114]. (iv) Thimerosal given in doses as low as 20 parts per billion affects the immune system of rats by altering dendritic cells (i.e., cells that initiate primary immune responses) which in turn activate T-cells causing the abnormal secretion of interleukin-6 (IL-6) [115]. Thus, environmental influences both *during* and *after *pregnancy can impact the immune system and the developing nervous system to play a role in producing neurodevelopmental disorders including ASDs. 

## 4. Conclusions and Future Perspectives

 With the increasing incidence of ASDs in the United States, it is most important to understand what has changed in our genes and environment that may contribute to these disorders. ASDs likely begin before or sometime after birth affecting the immune system and brain development. Because environmental insults can occur at different times during development, the variability in the neural and behavioral phenotype of ASDs is not unexpected. Changes in the brains of people with ASDs have been studied in fewer than 150 brains (see Table 1 that summarizes studies from 2003 to 2012). Most of these studies used semiquantitative methods with small sample sizes to analyze cell number and cell size; therefore, it is impossible to generalize the findings. However, the cerebellum has emerged as a region of interest in autism studies because of converging findings from human postmortem research, human neuroimaging studies, and animal models. The findings include (i) reductions in Purkinje cell number, the GABAergic output neurons of the cerebellum [116]; (ii) the cerebellum is often smaller in size in children with ASDs [21, 22]; (iii) tuberous sclerosis that results in autism is associated with tubers in the right cerebellum [75]; and (iv) an animal study that inserted the TSC1 mutation selectively into mouse Purkinje cells results in neurodegeneration of these neurons accompanied by an autistic behavioral phenotype [36]. Evidence appears to support cerebellar dysfunction, and more specifically, cerebellar GABA dysfunction as a contributor to the autism phenotype. Clinically, dysfunction of the right dentato-thalamo-frontal circuit may be a critical element in a subpopulation of ASDs.

Future studies will be needed to examine the details of brain neuropathology in postmortem brains from patients with ASDs, specifically in patients that exhibit *very homogeneous ASD behavioral phenotypes* and in those without epilepsy or mental retardation. Such studies should demonstrate more consistent neuropathological characteristics than have been observed to date in people with heterogeneous ASD phenotypes. These studies will only be possible when more postmortem tissues become available from both people with ASDs and age-matched typically-developing controls. Novel *in vivo* brain imaging techniques may be available sooner than postmortem tissue, and such techniques may allow new information about the neuropathology underlying ASDs.

It is interesting that several of the genes identified from human ASD genetic studies give rise to useful transgenic animal models of the disorder (see Figure 3). Many of these animal models exhibit both autism-like behavioral abnormalities and autistic-like neuropathology. For example, changes in the cerebellum, cortex, and hippocampus have been observed in transgenic animals with altered ASD-related genes. Furthermore, several of the abnormalities involve the GABA-containing neurons, the mTOR-signaling pathway, and the immune system.

The environment can also cause changes in brain development, as demonstrated in studies using thalidomide, valproic acid, and viral infection, as well as some maternal and paternal factors. Mercury toxicity from fish consumption has been linked to neurodevelopmental abnormalities, and the vaccine preservative thimerosal which contains ethylmercury has been suggested as a possible contributor to ASDs. However, the several epidemiological studies conducted to date have not found a link between thimerosal and autism. They have found a link, however, between thimerosal and tics [104, 105]. Because several studies indicate a relationship between tics and the cerebellum [117, 118], thimerosal-containing vaccines may play a role in such behavior as opposed to autism per se.

Thus, the heterogeneity observed in ASD neuropathology and behavior is likely due to multiple genetic and environmental factors that alter groups of GABAergic neurons in different regions of the brain (e.g., cerebellum, cortex and/or limbic system). In both the genetic and environmentally-linked animal models of ASDs, there are accompanying behavioral abnormalities involving sensory processing, seizure susceptibility, anxiety-like behavior, and motor abnormalities. Understanding how those affected with ASDs fit into various subgroups, with specific endophenotypes, will enable both a broader understanding of the causes of the disorders and providing insight into specific treatments.

## Figures and Tables

**Table 1:** Neuropathological findings in postmortem brains from autistic subjects from 2003 to 2012. Update of the table from Palmen et al. [19], which covered studies from 1980 to 2003.

Author and year	Journal	Sample size and features	Region of interest	Results
(1) Schumann and Amaral (2006) [37]	*J Neurosci. *	9 A; 10 C; all M 10–44 years	Amygdala-lateral, basal central nuclei	12% decrease in total amygdala neurons. Fourteen % decrease in neuron number in lateral nucleus.
(2) Van Kooten et al. (2008) [38]	*Brain *	7 A; 4 M, 3 F 10 C; 8 M, 2 F 3–50 years	Fusiform gyrus (FG) and visual cortex	Neurons are fewer and smaller in size (~10–20%) in FG in autism.
(3) Whitney et al. (2008) [28]	*Cerebellum *	6 A; 5 M, 1 F 4 C; 3 M, 1 F 17–54 years	Cerebellar Purkinje cells in crus II	Reduction in PC cell number in 3 of the 6 autism cases.
(4) Hutsler and Zhang (2010) [39]	*Brain Res *	10 A, 15 C 10–46 years	Frontal, parietal and temporal cortices	Increased spine density in layers 2 and 5, especially in temporal cortex.
(5) Courchesne et al. (2011) [40]	*JAMA. *	7 A; 6 C; all M 2–16 years	Dorsolateral and medial prefrontal cortices	More neurons (67%) in the prefrontal cortex in autism children with increased brain weight (17.6%)
(6) Santos et al. (2011) [41]	*Brain Res *	4 A; 2 M, 2 F 3 C; 2 M, 1 F 4–11 years	Fronto-insular cortex	58% more neurons in autism compared to controls.
(7) Jacot-Descombes et al. (2012) [42]	*Acta Neuropathol *	8 A; 6 M, 2 F 8 C; 7 M, 1 F 4–66 years	Dorsolateral prefrontal cortex	Smaller pyramidal neuronal size (18%) in Brodmann areas 44 and 45 in autism compared to controls. No change in cell number.

A: autism; C: control; M: male; F: female.

## References

[1] Bailey A, Phillips W, Rutter M (1996). Autism: towards an integration of clinical, genetic, neuropsychological, and neurobiological perspectives. *Journal of Child Psychology and Psychiatry and Allied Disciplines*.

[2] Amaral DG (2011). The promise and the pitfalls of autism research: an introductory note for new autism researchers. *Brain Research*.

[3] Kanner L (1943). Autistic disturbances of affective contact. *Child's Nervous System*.

[4] American Psychiatric Association (2000). *Diagnostic and Statistical Manual of Mental Disorders*.

[5] McCarthy J, Hemmings C, Kravariti E (2010). Challenging behavior and co-morbid psychopathology in adults with intellectual disability and autism spectrum disorders. *Research in Developmental Disabilities*.

[6] Fombonne E (2006). Autism and newborn encephalopathy. *Developmental Medicine and Child Neurology*.

[7] Tuchman R, Rapin I (2002). Epilepsy in autism. *Lancet Neurology*.

[8] Lecavalier L (2006). Behavioral and emotional problems in young people with pervasive developmental disorders: relative prevalence, effects of subject characteristics, and empirical classification. *Journal of Autism and Developmental Disorders*.

[9] Williams RS, Hauser SL, Purpura DP (1980). Autism and mental retardation. Neuropathologic studies performed in four retarded persons with autistic behavior. *Archives of Neurology*.

[10] Ritvo ER, Freeman BJ, Mason-Brothers A (1985). Concordance for the syndrome of autism in 40 pairs of afflicted twins. *American Journal of Psychiatry*.

[11] Ronald A, Happé F, Bolton P (2006). Genetic heterogeneity between the three components of the autism spectrum: a twin study. *Journal of the American Academy of Child and Adolescent Psychiatry*.

[12] Fombonne E (1999). The epidemiology of autism: a review. *Psychological Medicine*.

[13] Vorstman JAS, Morcus MEJ, Duijff SN (2006). The 22q11.2 deletion in children: high rate of autistic disorders and early onset of psychotic symptoms. *Journal of the American Academy of Child and Adolescent Psychiatry*.

[14] Moretti P, Zoghbi HY (2006). MeCP2 dysfunction in Rett syndrome and related disorders. *Current Opinion in Genetics and Development*.

[15] Jamain S, Quach H, Betancur C (2003). Mutations of the X-linked genes encoding neuroligins NLGN3 and NLGN4 are associated with autism. *Nature Genetics*.

[16] Strauss KA, Puffenberger EG, Huentelman MJ (2006). Recessive symptomatic focal epilepsy and mutant contactin-associated protein-like 2. *The New England Journal of Medicine*.

[17] Lossifov I, Ronemus M, Levy D, Wang Z (2012). De novo gene disruptions in children on the autistic spectrum. *Neuron*.

[18] Williams SC (2012). Genetics: searching for answers. *Nature*.

[19] Palmen SJMC, Van Engeland H, Hof PR, Schmitz C (2004). Neuropathological findings in autism. *Brain*.

[20] Strick PL, Dum RP, Fiez JA (2009). Cerebellum and nonmotor function. *Annual Review of Neuroscience*.

[21] Allen G (2005). Cerebellum in Autism. *Clinical Neuropsychiatry*.

[22] Allen G (2006). Cerebellar contributions to autism spectrum disorders. *Clinical Neuroscience Research*.

[23] Fatemi SH, Halt AR, Realmuto G (2002). Purkinje cell size is reduced in cerebellum of patients with autism. *Cellular and Molecular Neurobiology*.

[24] Kaufmann WE, Cooper KL, Mostofsky SH (2003). Specificity of cerebellar vermian abnormalities in autism: a quantitative magnetic resonance imaging study. *Journal of Child Neurology*.

[25] Bailey A, Luthert P, Dean A (1998). A clinicopathological study of autism. *Brain*.

[26] Bauman M, Kemper TL (1985). Histoanatomic observations of the brain in early infantile autism. *Neurology*.

[27] Lee M, Martin-Ruiz C, Graham A (2002). Nicotinic receptor abnormalities in the cerebellar cortex in autism. *Brain*.

[28] Whitney ER, Kemper TL, Bauman ML, Rosene DL, Blatt GJ (2008). Cerebellar Purkinje cells are reduced in a subpopulation of autistic brains: a stereological experiment using calbindin-D28k. *Cerebellum*.

[29] Yip J, Soghomonian JJ, Blatt GJ (2009). Decreased GAD65 mRNA levels in select subpopulations of neurons in the cerebellar dentate nuclei in autism: an in situ hybridization study. *Autism Research*.

[30] Kemper TL, Bauman M (1998). Neuropathology of infantile Autism. *Journal of Neuropathology and Experimental Neurology*.

[31] Ritvo ER, Freeman BJ, Scheibel AB (1986). Lower Purkinje cell counts in the cerebella of four autistic subjects: Initial findings of the UCLA-NSAC autopsy research report. *American Journal of Psychiatry*.

[32] Minshew NJ (2005). Neurologic apsects of autism. *Handbook of Autism and Pervasive Developmental Disorders*.

[33] Thach WT (1998). A role for the cerebellum in learning movement coordination. *Neurobiology of Learning and Memory*.

[34] Gowen E, Miall RC (2007). The cerebellum and motor dysfunction in neuropsychiatric disorders. *Cerebellum*.

[35] Amaral DG, Schumann CM, Nordahl CW (2008). Neuroanatomy of autism. *Trends in Neurosciences*.

[36] Tsai PT, Hull C, Chu Y, E Y (2012). Autistic-like behaviour and cerebellar dysfunction in Purkinje cell Tsc1 mutant mice. *Nature*.

[37] Schumann CM, Amaral DG (2006). Stereological analysis of amygdala neuron number in autism. *Journal of Neuroscience*.

[38] Van Kooten IAJ, Palmen SJMC, Von Cappeln P (2008). Neurons in the fusiform gyrus are fewer and smaller in autism. *Brain*.

[39] Hutsler JJ, Zhang H (2010). Increased dendritic spine densities on cortical projection neurons in autism spectrum disorders. *Brain Research*.

[40] Courchesne E, Mouton PR, Calhoun ME (2011). Neuron number and size in prefrontal cortex of children with autism. *Journal of the American Medical Association*.

[41] Santos M, Uppal N, Butti C (2011). von Economo neurons in autism: a stereologic study of the frontoinsular cortex in children. *Brain Research*.

[42] Jacot-Descombes S, Uppal N, Wicinski B (2012). Erratum to: decreased pyramidal neuron size in Brodmann areas 44 and 45 in patients with autism. *Acta Neuropathologica*.

[43] Nicolson R, DeVito TJ, Vidal CN (2006). Detection and mapping of hippocampal abnormalities in autism. *Psychiatry Research*.

[44] Kemper TL, Bauman ML (1993). The contribution of neuropathologic studies to the understanding of autism. *Neurologic Clinics*.

[45] Schumann CM, Barnes CC, Lord C, Courchesne E (2009). Amygdala enlargement in toddlers with autism related to severity of social and communication impairments. *Biological Psychiatry*.

[46] Schumann CM, Hamstra J, Goodlin-Jones BL (2004). The amygdala is enlarged in children but not adolescents with autism; the hippocampus is enlarged at all ages. *Journal of Neuroscience*.

[47] Aylward EH, Minshew NJ, Goldstein G (1999). MRI volumes of amygdala and hippocampus in non-mentally retarded autistic adolescents and adults. *Neurology*.

[48] Carper RA, Courchesne E (2005). Localized enlargement of the frontal cortex in early autism. *Biological Psychiatry*.

[49] Courchesne E, Pierce K (2005). Brain overgrowth in autism during a critical time in development: Implications for frontal pyramidal neuron and interneuron development and connectivity. *International Journal of Developmental Neuroscience*.

[50] Suzuki K, Ouchi Y, Nakamura K (2013). Microglial activation in young adults with autism spectrum disorder. *JAMA Psychiatry*.

[51] Hazlett HC, Poe MD, Gerig G, Smith RG, Piven J (2006). Cortical gray and white brain tissue volume in adolescents and adults with autism. *Biological Psychiatry*.

[52] Shukla DK, Keehn B, Müller R-A (2011). Tract-specific analyses of diffusion tensor imaging show widespread white matter compromise in autism spectrum disorder. *Journal of Child Psychology and Psychiatry and Allied Disciplines*.

[53] Noriuchi M, Kikuchi Y, Yoshiura T (2010). Altered white matter fractional anisotropy and social impairment in children with autism spectrum disorder. *Brain Research*.

[54] Irwin SA, Patel B, Idupulapati M (2001). Abnormal dendritic spine characteristics in the temporal and visual cortices of patients with fragile-X syndrome: a quantitative examination. *American Journal of Medical Genetics*.

[55] Argyropoulos A, Gilby KL, Hill-Yardin E (2013). Studying autism in rodent models: reconciling endophenotypes with comorbidities. *Frontiers in Human Neuroscience*.

[56] Chonchaiya W, Au J, Schneider A (2012). Increased prevalence of seizures in boys who were probands with the FMR1 premutation and co-morbid autism spectrum disorder. *Human Genetics*.

[57] Budimirovic DB, Kaufmann WE (2011). What can we learn about autism from studying fragile X syndrome?. *Developmental Neuroscience*.

[58] Tassone F, Choudhary NS, Durbin-Johnson B (2013). Identification of expanded alleles of the FMR1 gene in the CHhildhood Autism Risks from Genes and Environment (CHARGE) study. *Journal of Autism Developmental Disorders*.

[59] Bernardet M, Crusio WE (2006). *Fmr1* KO mice as a possible model of autistic features. *The Scientific World Journal*.

[60] Hays SA, Huber KM, Gibson JR (2011). Altered neocortical rhythmic activity states in *Fmr1* KO mice are due to enhanced mGluR5 signaling and involve changes in excitatory circuitry. *Journal of Neuroscience*.

[61] Peier AM, McIlwain KL, Kenneson A, Warren ST, Paylor R, Nelson DL (2000). (Over)correction of FMR1 deficiency with YAC transgenics: behavioral and physical features. *Human Molecular Genetics*.

[62] Grossman AW, Aldridge GM, Lee KJ (2010). Developmental characteristics of dendritic spines in the dentate gyrus of *Fmr1* knockout mice. *Brain Research*.

[63] Chahrour M, Zoghbi HY (2007). The story of Rett syndrome: from clinic to neurobiology. *Neuron*.

[64] Moy SS, Nadler JJ (2008). Advances in behavioral genetics: mouse models of autism. *Molecular Psychiatry*.

[65] Shahbazian MD, Young JI, Yuva-Paylor LA (2002). Mice with truncated MeCP2 recapitulate many Rett syndrome features and display hyperacetylation of histone H3. *Neuron*.

[66] Chao H-T, Chen H, Samaco RC (2010). Dysfunction in GABA signalling mediates autism-like stereotypies and Rett syndrome phenotypes. *Nature*.

[67] Yang T, Ramocki MB, Neul JL, W JL (2012). Overexpression of methyl-CpG binding protein 2 impairs T(H)1 responses. *Science Translational Medicine*.

[68] Comoletti D, De Jaco A, Jennings LL (2004). The Arg451Cys-neuroligin-3 mutation associated with autism reveals a defect in protein processing. *Journal of Neuroscience*.

[69] Yanagi K, Kaname T, Wakui K (2012). Identification of four novel synonymous substitutions in the X-linked genes *Neuroligin* 3 and *Neuroligin* 4X in Japanese patients with autistic spectrum disorder. *Autism Research Treatment*.

[70] Steinberg KM, Ramachandran D, Patel V (2012). Identification of rare X-linked neuroligin variants by massively parallel sequencing in males with autism spectrum disorder. *Molecular Autism*.

[71] Baudouin SJ, Gaudias J, Gerharz S (2012). Shared synaptic pathophysiology in syndromic and nonsyndromic rodent models of autism. *Science*.

[72] Yates JRW (2006). Tuberous sclerosis. *European Journal of Human Genetics*.

[73] Ertan G, Arulrajah S, Tekes A, Jordan L, Huisman TAGM (2010). Cerebellar abnormality in children and young adults with tuberous sclerosis complex: MR and diffusion weighted imaging findings. *Journal of Neuroradiology*.

[74] Weber AM, Egelhoff JC, Mckellop JM, Franz DN (2000). Autism and the cerebellum: evidence from tuberous sclerosis. *Journal of Autism and Developmental Disorders*.

[75] Eluvathingal TJ, Behen ME, Chugani HT (2006). Cerebellar lesions in tuberous sclerosis complex: neurobehavioral and neuroimaging correlates. *Journal of Child Neurology*.

[76] Chang S-C, Pauls DL, Lange C, Sasanfar R, Santangelo SL (2011). Common genetic variation in the GAD1 gene and the entire family of DLX homeobox genes and autism spectrum disorders. *American Journal of Medical Genetics B*.

[77] Stühmer T, Anderson SA, Ekker M, Rubenstein JLR (2002). Ectopic expression of the Dlx genes induces glutamic acid decarboxylase and Dlx expression. *Development*.

[78] Han S, Tai C, Westenbroek RE (2012). Autistic-like behaviour in Scn1a^+/-^ mice and rescue by enhanced GABA-mediated neurotransmission. *Nature*.

[79] Serajee FJ, Zhong H, Mahbubul Huq AHM (2006). Association of Reelin gene polymorphisms with autism. *Genomics*.

[80] Bonora E, Beyer KS, Lamb JA (2003). Analysis of reelin as a candidate gene for autism. *Molecular Psychiatry*.

[81] Guidotti A, Auta J, Davis JM (2000). Decrease in reelin and glutamic acid decarboxylase67 (GAD67) expression in schizophrenia and bipolar disorder: a postmortem brain study. *Archives of General Psychiatry*.

[82] Curran T, D’Arcangelo G (1998). Role of Reelin in the control of brain development. *Brain Research Reviews*.

[83] Hadj-Sahraoui N, Frédéric F, Delhaye-Bouchaud N, Mariani J (1996). Gender effect on Purkinje cell loss in the cerebellum of the heterozygous Reeler mouse. *Journal of Neurogenetics*.

[84] Gharani N, Benayed R, Mancuso V, Brzustowicz LM, Millonig JH (2004). Association of the homeobox transcription factor, ENGRAILED 2, 3, with autism spectrum disorder. *Molecular Psychiatry*.

[85] Butler MG, Dazouki MJ, Zhou X-P (2005). Subset of individuals with autism spectrum disorders and extreme macrocephaly associated with germline PTEN tumour suppressor gene mutations. *Journal of Medical Genetics*.

[86] Zhou J, Parada LF (2012). PTEN signaling in autism spectrum disorders. *Current Opinion in Neurobiology*.

[87] Zhou J, Blundell J, Ogawa S (2009). Pharmacological inhibition of mTORCl suppresses anatomical, cellular, and behavioral abnormalities in neural-specific PTEN knock-out mice. *Journal of Neuroscience*.

[88] Zhang XC, Piccini A, Myers MP (2012). Functional analysis of the protein phosphatase activity of PTEN. *Biochemistry Journal*.

[89] Stromland K, Nordin V, Miller M, Akerstrom B, Gillberg C (1994). Autism in thalidomide embryopathy: a population study. *Developmental Medicine and Child Neurology*.

[90] Hranilovic D, Bujas-Petkovic Z, Vragovic R, Vuk T, Hock K, Jernej B (2007). Hyperserotonemia in adults with autistic disorder. *Journal of Autism and Developmental Disorders*.

[91] Piven J, Tsai G, Nehme E, Coyle JT, Chase GA, Folstein SE (1991). Platelet serotonin, a possible marker for familial autism. *Journal of Autism and Developmental Disorders*.

[92] Williams G, King J, Cunningham M, Stephan M, Kerr B, Hersh JH (2001). Fetal valproate syndrome and autism: additional evidence of an association. *Developmental Medicine and Child Neurology*.

[93] Ingram JL, Peckham SM, Tisdale B, Rodier PM (2000). Prenatal exposure of rats to valproic acid reproduces the cerebellar anomalies associated with autism. *Neurotoxicology and Teratology*.

[94] Narita N, Kato M, Tazoe M, Miyazaki K, Narita M, Okado N (2002). Increased monoamine concentration in the brain and blood of fetal thalidomide- and valproic acid-exposed rat: putative animal models for autism. *Pediatric Research*.

[95] Grandjean P, Budtz-Jørgensen E, White RF (1999). Methylmercury exposure biomarkers as indicators of neurotoxicity in children aged 7 years. *American Journal of Epidemiology*.

[96] Falluel-Morel A, Sokolowski K, Sisti HM, Zhou X, Shors TJ, DiCicco-Bloom E (2007). Developmental mercury exposure elicits acute hippocampal cell death, reductions in neurogenesis, and severe learning deficits during puberty. *Journal of Neurochemistry*.

[97] Davidson PW, Cory-Slechta DA, Thurston SW (2011). Fish consumption and prenatal methylmercury exposure: cognitive and behavioral outcomes in the main cohort at 17 years from the Seychelles child development study. *NeuroToxicology*.

[98] Hviid A, Stellfeld M, Wohlfahrt J, Melbye M (2003). Association between thimerosal-containing vaccine and Autism. *Journal of the American Medical Association*.

[99] Thompson WW, Price C, Goodson B (2007). Early thimerosal exposure and neuropsychological outcomes at 7 to 10 years. *The New England Journal of Medicine*.

[100] Madsen KM, Lauritsen MB, Pedersen CB (2003). Thimerosal and the occurrence of autism: negative ecological evidence from Danish population-based data. *Pediatrics*.

[101] Delong G (2011). A positive association found between autism prevalence and childhood vaccination uptake across the U.S. population. *Journal of Toxicology and Environmental Health A*.

[102] Barile JP, Kuperminc GP, Weintraub ES, Mink JW, Thompson WW (2012). Thimerosal exposure in early life and neuropsychological outcomes 7-10 years later. *Journal of Pediatric Psychology*.

[103] Kurlan R, McDermott MP, Deeley C (2001). Prevalence of tics in schoolchildren and association with placement in special education. *Neurology*.

[104] Verstraeten T, Davis RL, DeStefano F (2003). Safety of thimerosal-containing vaccines: a two-phased study of computerized health maintenance organization databases. *Pediatrics*.

[105] Tozzi AE, Bisiacchi P, Tarantino V (2009). Neuropsychological performance 10 years after immunization in infancy with thimerosal-containing vaccines. *Pediatrics*.

[106] Atladóttir HÓ, Thorsen P, Østergaard L (2010). Maternal infection requiring hospitalization during pregnancy and autism spectrum disorders. *Journal of Autism and Developmental Disorders*.

[107] Shi L, Smith SEP, Malkova N, Tse D, Su Y, Patterson PH (2009). Activation of the maternal immune system alters cerebellar development in the offspring. *Brain, Behavior, and Immunity*.

[108] Hsiao EY, McBride SW, Chow J (2012). Modeling an autism risk factor in mice leads to permanent immune dysregulation. *Proceedings of the National Academy of Sciences of the United States of America*.

[109] Garay PA, Hsiao EY, Patterson PH, McAllister AK (2013). Maternal immune activation causes age- and region-specific changes in brain cytokines in offspring throughout development. *Brain Behavior and Immunity*.

[110] Kong A, Frigge ML, Masson G, S G (2012). Rate of de novo mutations and the importance of father's age to disease risk. *Nature*.

[111] Pearce BD (2003). Modeling the role of infections in the etiology of mental illness. *Clinical Neuroscience Research*.

[112] Schneider T, Roman A, Basta-Kaim A (2008). Gender-specific behavioral and immunological alterations in an animal model of autism induced by prenatal exposure to valproic acid. *Psychoneuroendocrinology*.

[113] Libbey JE, Sweeten TL, McMahon WM, Fujinami RS (2005). Autistic disorder and viral infections. *Journal of NeuroVirology*.

[114] Patterson PH (2011). Maternal infection and immune involvement in autism. *Trends in Molecular Medicine*.

[115] Goth SR, Chu RA, Gregg JP, Cherednichenko G, Pessah IN (2006). Uncoupling of ATP-mediated calcium signaling and dysregulated interleukin-6 secretion in dendritic cells by nanomolar thimerosal. *Environmental Health Perspectives*.

[116] Pizzarelli R, Cherubini E (2011). Alterations of GABAergic signaling in autism spectrum disorders. *Neural Plasticity*.

[117] Lerner A, Bagic A, Boudreau EA (2007). Neuroimaging of neuronal circuits involved in tic generation in patients with Tourette syndrome. *Neurology*.

[118] Stern E, Silbersweig DA, Chee K-Y (2000). Functional neuroanatomy of tics in Tourette syndrome. *Archives of General Psychiatry*.

